# Success After Endodontic Microsurgery: A Critical, Time- and Modality-Dependent Interpretation

**DOI:** 10.3390/dj14080502

**Published:** 2026-08-08

**Authors:** Hadar Better, Thabet Asbi, Gavriel Chaushu, Keren Better, Joshua Moshonov, Doron Haim

**Affiliations:** 1 Medical & Quality Assurance Department, Maccabi-Dent, Tel Aviv 6801298, Israel; 2 Research and Innovation Department, Maccabi-Dent, Tel Aviv 6801298, Israel; 3 Department of Periodontology, Rambam Health Care Campus, Haifa 3109601, Israel; 4 Department of Oral and Maxillofacial Surgery, Rabin Medical Center, Petah Tikva 4941492, Israel; 5 Department of Information and Computing Sciences, Utrecht University, 3584 CC Utrecht, The Netherlands; 6 Health Management Department, Faculty of Health Sciences, Ariel University, Ariel 40700, Israel

**Keywords:** endodontic microsurgery, interpretive review, outcome, periapical radiography, periapical healing, cone-beam computed tomography

## Abstract

**Background/Objectives:** Meta-analyses of endodontic microsurgery (EMS) report pooled success rates of 90–94%, which is now a reference point for clinical decisions. These estimates are often applied beyond the follow-up intervals and imaging conditions in which they were derived. This review defines the temporal and imaging boundaries within which such a rate can be applied with confidence. **Methods:** We reviewed the core meta-analyses establishing these benchmarks, together with primary studies comparing periapical radiography (PA) and cone-beam computed tomography (CBCT) within the same cohort or across follow-up intervals. Sources from MEDLINE, Embase, and the Cochrane Library were selected purposively, not by systematic screening. **Results:** Reported success varied with both imaging modality and follow-up time. In within-cohort comparisons, PA consistently yielded higher success than CBCT, being less sensitive to residual cancellous defects; in one cohort, CBCT found a defect in 28% of sites PA had judged healed. A long-term meta-analysis reported 91.3% success in randomized trials but 78.4% in prospective cohorts over 2 to 13 years, with 5 to 25% of early successes later reverting. **Conclusions:** Success after EMS is neither temporally stable nor modality-neutral. Meta-analytic estimates are valid within their stated follow-up and imaging context; applying them outside that context risks misclassification with direct consequences for clinical decision-making.

## 1. Introduction

Meta-analyses of endodontic microsurgery (EMS) report pooled success rates of 90–94% [1,2,3], and this figure has become the reference point for clinical decisions about whether to keep a tooth, when to restore it, and whether to re-enter. The number is often used as if it were a fixed property of the procedure. In fact, it is a measurement taken at a particular moment, with a particular imaging method, and both conditions are built into the number. These distinctions matter because clinicians act on them before healing is complete. A restoration is placed, a prosthesis is loaded, or surgery is repeated largely on the judgment that the site has healed, and that judgment often rests on a single reported success rate. If the rate is read without the follow-up interval and imaging method behind it, the same radiograph can justify opposite decisions: restoring a tooth that later fails or re-entering one that would have settled on its own. Defining when such a rate applies is therefore a practical clinical question, not only a methodological one.

Healing after surgery is not static. Removing the chronically inflamed periapical tissue changes the kinetics of repair: once the activated macrophages and bone-resorbing cytokines that sustain the lesion are cleared, healing proceeds faster [4,5]. Kvist and Reit [6] showed the clinical counterpart directly: surgically treated lesions healed faster, with a significant advantage at 12 months that had disappeared by 48 months. They attributed the convergence to two processes: slower healing in the nonsurgical group and a number of late failures in the surgical group, cases counted as healed at one year that had relapsed by four. Either way, the rate measured early and the rate measured late describe the same cohort at different points on a moving curve.

A more recent meta-analysis [7] makes the same point in modern terms. Restricting inclusion to studies with two or more years of follow-up, pooled success was 91.3% across randomized trials but 78.4% across prospective cohorts, and 5 to 25% of teeth judged healed in the short term had reverted when reassessed at three years or longer. Whether one year of follow-up is enough to predict the long-term result remains debated, and some evidence suggests it often is. But prediction is not the same as measurement, and the debate itself assumes a single imaging method.

That assumption is the second problem. Periapical radiography (PA) is two-dimensional and misses residual defects contained within cancellous bone, whereas cone-beam computed tomography (CBCT) detects them [8]. In a paired cohort, CBCT found a remaining defect in 28% of sites that the radiograph had already classified as healed [9]. This discrepancy is systematic rather than random, and it narrows over time as remodeling progresses: in a five-year trial, the difference between radiographic and CBCT success fell from 25% at one year to 12.5% at five [10]. Whether a case is counted as a success depends on which instrument looked and when.

Taken together, the dependence of success on both follow-up time and imaging modality means that a single pooled rate cannot be carried across contexts without losing its meaning. A 12-month, radiograph-based rate rests on one set of assumptions; a five-year CBCT rate rests on another. This review takes the established meta-analytic evidence as its starting point and sets out to define the temporal and imaging boundaries within which a reported EMS success rate can be applied with confidence. Reviews have addressed the time-dependence and the imaging-dependence of reported outcomes separately; the contribution here is to treat the two jointly and to translate their interaction into explicit boundaries for applying a reported success rate in clinical practice.

## 2. Methods

This is a critical, interpretive review rather than a systematic review, and it does not claim exhaustive or reproducible identification of the literature. We identified the meta-analyses that established the benchmark EMS success rates, together with primary studies that either compared imaging modalities within the same cohort or reported outcomes across more than one follow-up interval. The relevant literature was identified through the major bibliographic databases, citation tracking from key articles, and the authors’ clinical and surgical experience, rather than through a predefined search and screening protocol. Selection was purposive: a source was included when it bore directly on how reported success depends on follow-up time or imaging modality. A study met the modality criterion if it compared periapical radiography with CBCT in the same postoperative cohort and the time criterion if it reported endodontic microsurgery outcomes at more than one follow-up interval or at long-term follow-up. Studies were set aside when they addressed only nonsurgical or primary endodontic treatment or diagnostic accuracy in untreated apical periodontitis rather than postoperative healing. No minimum follow-up threshold and no date limit were applied. Because the review examines how classification changes over time, the evidence deliberately spans early postoperative assessment through follow-up of several years, across sources published between 1987 and 2025. Related systematic reviews and meta-analyses that were considered during scoping but fall outside this specific question are listed, with the reason for non-selection, in Table S1. These reviews are placed in the Supplementary Material because they document the boundary of the review question, that is, what was considered and set aside, rather than forming part of the evidence used to reach the conclusions.

Two layers of evidence are distinguished throughout. The first comprises the core meta-analyses used as reference points for the headline figures. The second comprises primary studies that illustrate how outcome classification shifts with time or with the imaging method used. Where studies permitted a direct within-cohort comparison of periapical radiography and CBCT, the direction of the difference is reported descriptively; every such study found radiography to yield the higher success classification, consistent with the lower sensitivity of two-dimensional imaging to residual cancellous defects. No formal hypothesis test was applied, since the direction of the modality effect follows from the known properties of the two methods rather than from a sampling process.

## 3. Results

### 3.1. Core Meta-Analyses (Reference Layer)

Three evidence syntheses [1,2,3] anchored the headline success figures of 90–94% (Table 1). Their pooled estimates were derived predominantly from outcomes assessed by periapical radiography at follow-up intervals of 6 to 12 months, with longer intervals represented unevenly (Table 2). None reported a single rate that held constant across both imaging method and follow-up duration.

### 3.2. Within-Cohort Imaging Comparisons (Modality Layer)

Four primary studies compared periapical radiography and CBCT directly within the same postoperative cohort (Christiansen et al. [9], von Arx et al. [14], Schloss et al. [15], and Dhamija et al. [10]; Table 3). In each, radiography returned the higher success classification. Table 3 summarizes these studies, their imaging comparisons, and the interpretive boundary each supports. The direction is consistent and follows from a known property of the two methods: two-dimensional radiography fails to detect residual defects confined within cancellous bone, which CBCT resolves. In the paired cohort reported by Christiansen et al., the two methods agreed on defect status in roughly 67% of sites (n = 52), and in 28% of sites that radiography had classified as healed, CBCT identified a residual defect. These figures describe a single cohort and illustrate the modality effect rather than providing a pooled estimate. Two systematic reviews assessing the same question across independent samples (Rosen et al. [16]; Sharma et al. [11]) report the same direction, and a histologic-validation study (Kruse et al. [17]) confirms that radiographic classification, by either modality, does not map directly onto biological status, since a substantial proportion of lesions judged unresolved on imaging showed no inflammation on biopsy. Together, these provide three layers of corroboration for the modality effect: paired within-cohort comparison, independent systematic review, and histologic reference.

### 3.3. Outcomes Across Follow-Up Intervals (Time Layer)

**Figure 1:**
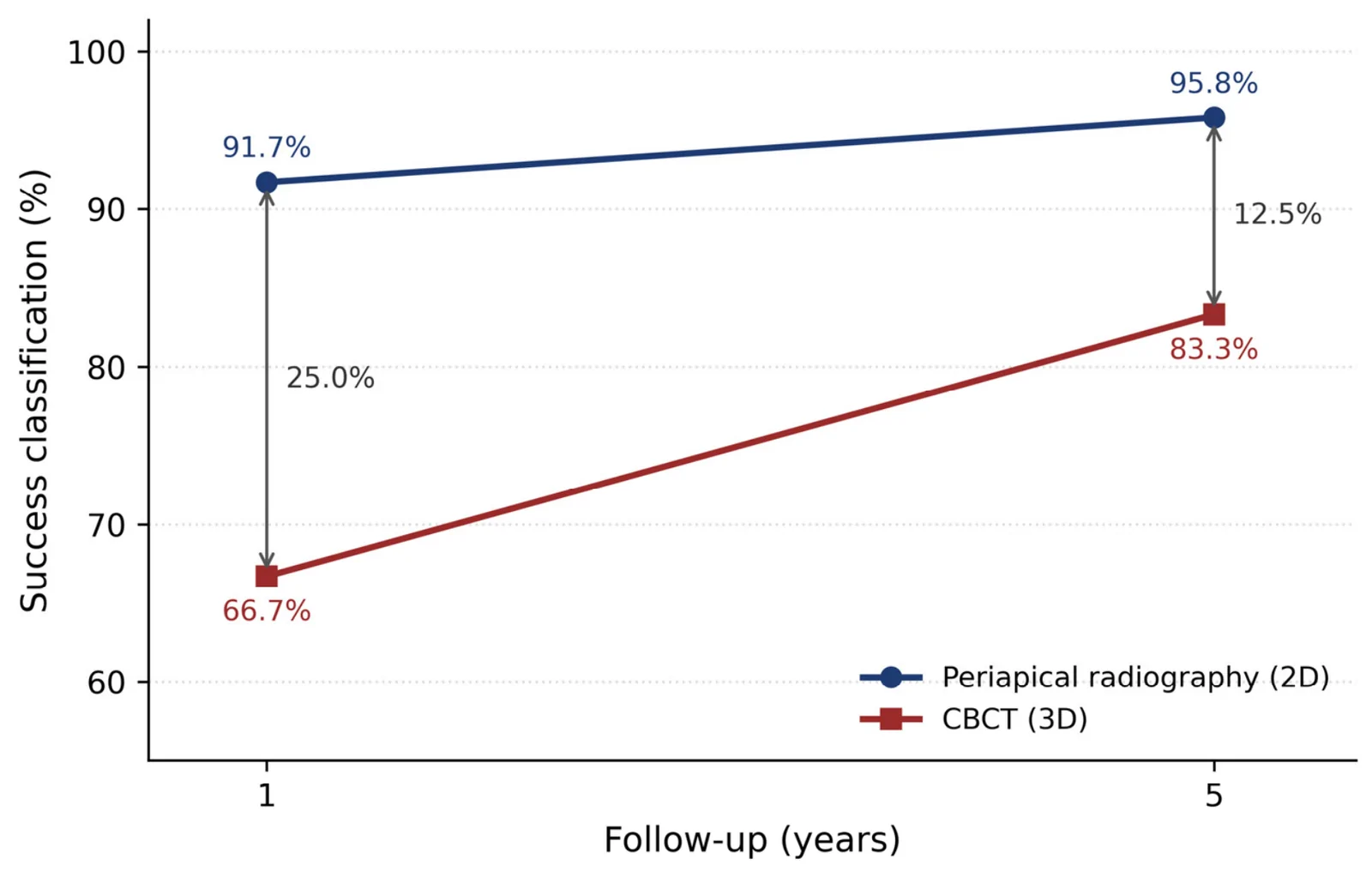
Time- and modality-dependent success rates after endodontic microsurgery. Success rates assessed using two-dimensional periapical radiography (2D) and three-dimensional cone-beam computed tomography (3D) at 1- and 5-year follow-up. Data from Dhamija et al. [10]; success is reported per patient for the same 24 patients (44 teeth) assessed at both follow-ups. The absolute modality gap narrows from 25.0% to 12.5% over time, reflecting ongoing remodeling while a modality difference persists.

**Figure 2:**
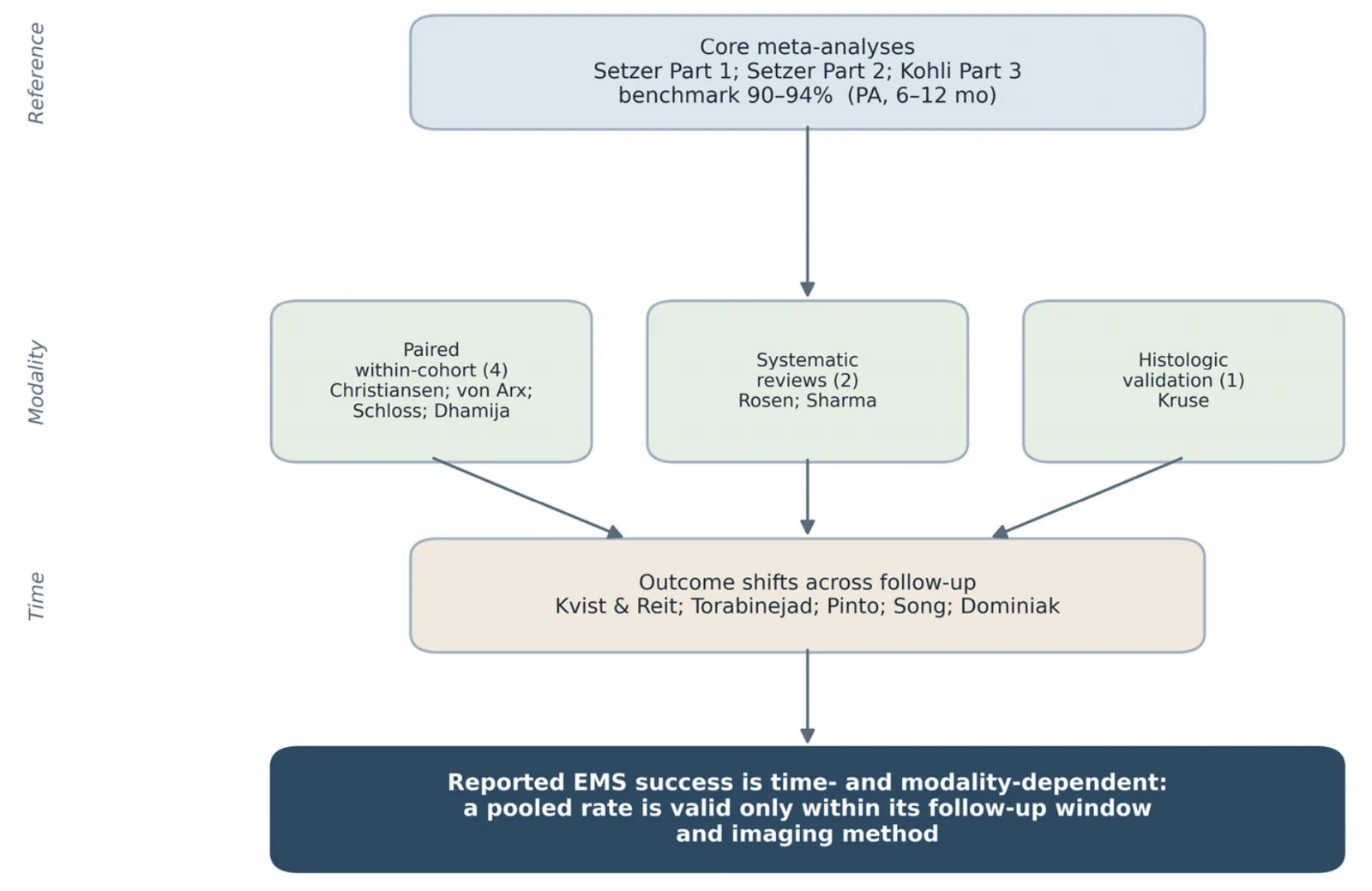
Evidence structure of this interpretive review. The reference layer comprises the three core meta-analyses that establish the 90–94% benchmark. The modality layer draws on three independent forms of evidence: four paired within-cohort comparisons of periapical radiography and CBCT, two systematic reviews, and one histologic-validation study. The time layer assembles studies documenting reclassification of outcomes across follow-up intervals. The diagram shows how these layers converge on the central interpretation rather than depicting a systematic screening or selection process; this review does not apply systematic-review methodology [1,2,3,6,7,9,10,11,12,14,15,16,17,18,19].

Beyond the imaging comparison, several cohorts reported that healing classification shifts with time. Cases counted as successful under short-term radiographic assessment were reclassified at later intervals in both directions: some lesions judged unresolved at one year had healed by five, while a smaller number counted as healed early relapsed later. The five-year trial of through-and-through lesions showed the gap between radiographic and CBCT success narrowing from 25% at one year to 12.5% at five as remodeling progressed (Figure 1), illustrating that the modality effect and the time effect are not independent. The overall structure of the evidence base examined in this review is summarized in Figure 2.

## 4. Discussion

Outcome classification after EMS depends on both how healing is imaged and when it is assessed. In the paired cohort of Christiansen et al. [9], periapical radiography and CBCT agreed on defect status in roughly 67% of sites, and CBCT identified a residual defect in 28% of sites that radiography had classified as healed. The same direction held in every within-cohort comparison available: radiography returns the higher success rate, not because healing is more complete but because it is less sensitive to residual defects confined within cancellous bone. Since the same patients were assessed by both methods, the disagreement reflects detection sensitivity rather than any difference in healing. Detecting a defect is not, however, the same as detecting active disease: Kruse et al. [17] found that many defects still visible on CBCT corresponded to non-inflammatory tissue on biopsy, so neither modality maps cleanly onto biological status.

The time-dependent dimension is equally clear. The core meta-analyses report outcomes at fixed thresholds, most often 6 or 12 months, which permit consistent pooling but do not capture the full healing trajectory. Healing continues well beyond these windows, and classification shifts accordingly. Kvist and Reit [6] showed that the advantage of surgical over nonsurgical retreatment, significant at 12 months, had vanished by 48 months, as the slower-healing group caught up and a few early successes relapsed. The more recent meta-analysis of long-term EMS [7] makes the same point at scale: pooled success was 91.3% across randomized trials but fell to 78.4% across prospective cohorts followed for t 2 to 13 years, with 5 to 25% of early successes reverting on longer observation. A success rate anchored to one time point cannot be treated as a stable estimate.

Clinical decisions about definitive restoration, prosthetic loading, or surgical re-entry often arise before radiographic healing is confirmed. These decision points are rarely studied directly; in practice, clinicians proceed to definitive restoration once symptoms have resolved and radiographic healing appears adequate, often guided by consensus follow-up thresholds rather than by a measure of stability over time [1,2,3,7,11]. Clinical readiness here refers to the threshold at which a clinician judges a site suitable for such time-sensitive management. Since readiness is seldom measured directly in the available studies, reported success rates should not be used as its surrogate. In the absence of a validated stability measure, we suggest that readiness be judged not from a single success figure but from the convergence of three observations: resolution of clinical symptoms; radiographic stability assessed across two successive time points rather than a single snapshot; and, where CBCT is used, the absence of an enlarging defect. This operational triad does not constitute a standardized outcome, but it offers a transparent interim basis for decision-making until one is validated. CBCT use has been shown to shift clinical decisions toward more invasive options [21], which underscores the need for interpretive caution when imaging findings diverge from published PA-based benchmarks. The practical guidance summarized in the following subsection follows directly from the two dependencies established here: the items addressing modality and follow-up operationalize the imaging and time boundaries, while the remaining items address the interpretive caution required when imaging and symptoms diverge. This guidance is deliberately conditional rather than prescriptive, since the evidence supports the direction of these effects but not fixed threshold values; it reflects the authors’ interpretive framework and is not a formal clinical guideline.

### Clinical Significance

1. Always cite the follow-up window alongside the success rate: “94% success at 12 months” conveys more than “94% success”.
2. Specify the imaging modality used; PA-based and CBCT-based rates are not interchangeable.
3. Interpret early follow-up classifications as provisional: healing at 6 months does not confirm stability at 24 months.
4. When CBCT is used at follow-up, anticipate possible reclassification relative to published PA-based benchmarks.
5. Interpret imaging discordance in light of clinical symptoms and postoperative time rather than treating it as an automatic contradiction of published success rates [9,10,11,14,15,17].
6. Consider symptom resolution and patient-reported function alongside imaging findings when assessing readiness for definitive treatment.
7. When CBCT follow-up is planned, consider deferring imaging beyond 6 months post-surgery to reduce the risk of misclassifying incomplete, ongoing healing as failure; discuss this timing rationale explicitly with the patient.

Clinicians should view reported success rates as time-bound, modality-specific snapshots of healing status, not as guarantees of long-term stability or clinical readiness.

The core meta-analytic series [1,2,3] establishes the PA-based success benchmarks from which this review takes its starting point. Sharma et al. [11] provide the modality comparison anchor, directly evaluating whether outcome classification varies by imaging method. Torabinejad et al. [12] and Kang et al. [22] extend the temporal frame, with Torabinejad et al. demonstrating that comparative conclusions between surgical and nonsurgical treatment can reverse across follow-up strata. Pinto et al. [7] offer independent corroboration from a separate author group, reducing reliance on a single research series.

Recent evidence continues to report high EMS success rates. Ko et al. [23] found 89% success for EMS versus 78% for single implants. These figures are clinically meaningful, but their value depends on knowing when and how healing was measured. The temporal and modality boundaries identified in this review apply equally to recent comparative data.

## 5. Limitations

Several limitations should be acknowledged. As an interpretive review, it rests on a purposive selection of evidence guided by explicit criteria but shaped by the authors’ conceptual framework, and it does not claim the exhaustive, reproducible ascertainment of a systematic review. Several of the benchmark estimates derive from a single meta-analytic series (the Setzer and Kohli group), and the within-cohort modality comparisons rest on a small number of cohorts, so larger and longer-term datasets using CBCT are needed to confirm and quantify these effects. Because the source studies differ in their outcome criteria and imaging methods, the modality and time effects are described in terms of direction rather than pooled quantitatively. Direct measures of the point at which a site becomes suitable for definitive treatment were largely absent from the reviewed literature, and patient-reported outcomes linked to specific follow-up intervals and imaging modalities remain underreported, which limits how precisely these thresholds can be defined.

Future research should prioritize longitudinal CBCT datasets that track healing trajectories beyond 12 months, studies that directly measure clinical decision points rather than retrospective radiographic classifications, and the development of standardized criteria for clinical readiness that integrate imaging findings with patient-reported outcomes.

## 6. Conclusions

Reported success after endodontic microsurgery is a function of both the follow-up interval and the imaging modality used to assess healing. Meta-analytic estimates remain valid and clinically useful within the temporal and imaging contexts in which they were derived; they cannot, however, serve as modality-neutral or time-independent markers of clinical readiness. When planning definitive restoration or comparing treatment options, clinicians should know not only the reported success rate but also when it was measured and by which imaging method. None of this diminishes the published estimates. They remain a sound summary of what EMS can achieve, as long as they are read as answers to a specific question rather than as a fixed figure. The rate tells us what was observed; the follow-up interval and the imaging method help tell us what it means. Until healing over time can be measured more directly, a reported success rate may be best treated as provisional, valid for the moment and the method behind it.

## Figures and Tables

**Table 1:** Evidence syntheses reviewed as anchors.

ID	Meta-Analysis	Journal	Scope/Comparison	Assessment Modality	Min Follow-Up	Role in This Paper	Anchor Logic
M1	Setzer et al. [1] (Part 1)	J. Endod.	TRS vs. EMS	PA + clinical	≥6 months	Core Anchor—technique era	Technique series
M2	Setzer et al. [2] (Part 2)	J. Endod.	High vs. lower magnification in EMS	PA + clinical	≥12 months	Core Anchor—magnification	Technique series
M3	Kohli et al. [3] (Part 3)	J. Endod.	Root-end filling materials in EMS	PA + clinical	≥12 months (often ≥1 y)	Core Anchor—materials	Technique series
M4	Sharma et al. [11]	Imaging Sci. Dent.	Healing assessment: PA vs. CBCT	PA vs. CBCT	Variable	Modality Boundary Anchor	Modality
M5	Pinto et al. [7]	Medicina	Long-term prognosis of EMS	Predominantly PA	Long-term focus	Independent corroboration (descriptive)	External corroboration
M6	Torabinejad et al. [12]	J. Endod. (2009)	Nonsurgical retreatment vs. endodontic surgery (indirect comparison)	Predominantly periapical radiography + clinical assessment	≥2 y (mean), stratified 2–4 y and 4–6 y	Boundary anchor—time-trajectory/comparative framing	Demonstrates reversal of apparent advantage across follow-up windows

TRS = traditional root-end surgery; EMS = endodontic microsurgery; PA = periapical radiography; CBCT = cone-beam computed tomography.

**Table 2:** Outcome definitions and follow-up thresholds in core meta-analyses.

Meta-Analysis	Outcome Definition	Follow-Up Thresholds	Assessment Modality Context	Interpretive Boundary
Setzer et al. [1]	Radiographic healing classified via PA using Rud/Molven-type criteria [13]; clinical success by absence of symptoms.	Minimum follow-up ≥6 months.	PA radiography + clinical assessment.	Success reflects status at the reported follow-up; avoid time-independent or early-decision inference.
Setzer et al. [2]	PA-based radiographic healing criteria; clinical success by symptom absence.	Minimum follow-up ≥12 months.	PA radiography + clinical assessment.	Later windows still do not describe trajectories or time to downstream decisions.
Kohli et al. [3]	Radiographic healing on PA, plus clinical status and failure events.	Minimum follow-up ≥12 months (often ≥1 year).	PA radiography + clinical assessment.	Do not treat PA-based success as modality-neutral when CBCT adjudicates healing.
Sharma et al. [11]	Healing classifications compared conventional radiography and CBCT across studies (definitions vary).	Variable across included studies.	Explicitly PA vs. CBCT.	Outcome labels depend on modality; compare across modalities with caution.
Pinto et al. [7]	Radiographic and clinical success (predominantly PA-based criteria).	Long-term follow-up emphasis (as defined in included studies).	PA radiography + clinical assessment.	Supports time dependence but does not answer early decision questions.
Torabinejad et al. [12]	Success defined as complete + incomplete healing (Rud-derived categories); failure includes uncertain + unsatisfactory.	≥2 years mean follow-up; reported by recall strata (2–4 y, 4–6 y; limited data beyond 6 y).	Outcomes extracted from studies using clinical ± periapical radiographic assessment (era-heterogeneous).	Time window can reverse comparative conclusions; early success is not a stable endpoint.

**Table 3:** Primary studies illustrating time- and modality-dependent outcome classification.

Study	Design	Modality	Time Points	Main Result	Interpretive Boundary
Christiansen et al. [9]	Prospective clinical follow-up	PA vs. CBCT	1 week, 12 months	At 12 months (n = 52), agreement on defect presence/absence was 67%; CBCT detected a remaining defect in 28% of sites where PA showed none.	Modality-dependent defect detection at 12 months; CBCT identifies residual defects missed by PA. Reclassification should be interpreted with caution rather than as automatic failure.
von Arx et al. [14]	Cohort follow-up	PA (2D) vs. CBCT (3D)	12 months	Partial agreement between 2D and 3D outcome classifications.	‘Healed’ on PA does not automatically map to ‘healed’ on CBCT; modality should be stated when interpreting success.
Schloss et al. [15]	Cohort outcome assessment	PA vs. CBCT volumes	≥12 months	Outcome classification differed between 2D and 3D assessments in a clinically relevant subset.	Modality-driven reclassification persists at later follow-up; pooled PA-based rates are not modality-neutral.
Kruse et al. [17]	Diagnostic validity study (postoperative)	PA vs. CBCT	Post-surgical persistent lesion evaluation	CBCT showed higher diagnostic validity for persistent lesions than PA. Histologically, 42% of CBCT-classified failures showed no inflammation on biopsy; 75% of uncertain healing cases were non-inflammatory.	Radiographic classification does not map directly onto biological status and should be interpreted in the clinical context.
Song et al. [18]	Longitudinal observational follow-up	PA + clinical	Short-term vs. later follow-up	Some cases labeled successful at short-term follow-up changed classification later.	Short-term success should be interpreted as provisional with respect to longer-term stability.
Dominiak et al. [19]	Prospective follow-up	PA	6, 12 months	Healing continued between 6 and 12 months.	Early windows capture direction of healing rather than stabilization.
Peñarrocha et al. [20]	Prospective cohort	Clinical + radiographic	12 months	Preoperative symptoms associated with postoperative outcome.	Symptom status adds clinical context; radiographic success alone does not fully specify postoperative clinical state.
Dhamija et al. [10]	RCT with long-term follow-up	2D (PA) vs. 3D (CBCT)	1 year, 5 years	3D healing improved from 66.7% at 1 year to 83.3% at 5 years; 2D and 3D assessments showed discordant classifications.	Both time-dependent improvement and modality-dependent reclassification observed in same cohort.
Rosen et al. [16]	Systematic review	PA vs. CBCT	Variable (included studies)	PA success 90% (95% CI 86–92%) vs. CBCT 35% (95% CI 17–57%); uncertain healing 48% on CBCT vs. 4% on PA.	Modality-dependent success classification; PA-based rates substantially overestimate success relative to CBCT.

## Data Availability

No new data were created or analyzed in this study. Data sharing is not applicable to this article.

## Supplementary Materials

The following supporting information can be downloaded at https://www.mdpi.com/article/10.3390/dj14080502/s1. Table S1: Related systematic reviews and meta-analyses considered but not used as anchors in this interpretive review, with the reason for non-selection [24,25,26,27,28,29,30,31,32,33].
