# Supplementary material for: Success After Endodontic Microsurgery: A Critical, Time- and Modality-Dependent Interpretation
Source: Dent J (Basel). 2026 Aug 8;14(8):502. doi: 10.3390/dj14080502 (PMC13510885; doi:10.3390/dj14080502)
Supplement: Supplementary file 1 [file dentistry-14-00502-s001.zip › dentistry-4435633-supplementary.pdf]

Nonsurgical Supplementary Table S1. Related systematic reviews and meta-analyses considered but not used as anchors in this interpretive review.

These reviews were considered during scoping but fall outside the specific question of this review: the postoperative classification of endodontic microsurgery (EMS) healing by imaging modality and follow-up time. They are listed for transparency. This table reflects the authors' scoping of the related literature and is not a systematic screening log.

Citation	Primary focus	Reason not used as an anchor
Brochado Martins et al., 2025. [24] J. Endod. doi:10.1016/j.joen.2025.03.004	CBCT-assessed outcomes of primary endodontic treatment and retreatment	Addresses primary/nonsurgical treatment, not postoperative EMS healing
Ramis-Alario et al., 2021. [25] Oral Surg. Oral Med. Oral Pathol. Oral Radiol. doi:10.1016/j.oooo.2021.07.002	Diagnostic efficacy of 2D radiography vs. CBCT in persistent apical disease	Addresses diagnostic accuracy, not postoperative EMS healing assessment
Kruse et al., 2015. [26] Int. Endod. J. doi:10.1111/iej.12388	CBCT and periapical lesions: diagnostic efficacy systematic review	Addresses diagnostic accuracy, not postoperative EMS outcome classification
Aminoshariae et al., 2018. [27] J. Endod. doi:10.1016/j.joen.2018.08.006	CBCT vs. intraoral radiography in endodontic outcome studies	Includes nonsurgical endodontic outcomes; not specific to postoperative EMS healing
Leonardi Dutra et al., 2016. [28] J. Endod. doi:10.1016/j.joen.2015.12.015	Diagnostic accuracy of CBCT vs. conventional radiography for apical periodontitis	Addresses diagnostic accuracy, not postoperative EMS outcome classification
Abesi & Golikani, 2023. [29] Pol. J. Radiol. doi:10.5114/pjr.2023.134035	Diagnostic performance of CBCT for apical periodontitis	Addresses diagnostic accuracy, not postoperative EMS outcome classification
Hilmi et al., 2023. [30] Int. Endod. J. doi:10.1111/iej.13921	Efficacy of imaging techniques for diagnosis of apical periodontitis	Addresses diagnostic accuracy, not postoperative EMS outcome classification
Mostafapoor & Hemmatian, 2022. [31] Oral Radiol. doi:10.1007/s11282-021-00549-z	Accuracy of CBCT for apical periodontitis	Addresses diagnostic accuracy, not postoperative EMS outcome classification
Del Fabbro et al., 2016. [32] Cochrane Database Syst. Rev. doi:10.1002/14651858.CD005511.pub3	Endodontic procedures for retreatment of periapical lesions	Addresses retreatment procedures broadly; does not specifically assess postoperative EMS outcomes
Yapp et al., 2021. [33] J. Med. Imaging. doi:10.1117/1.JMI.8.4.041205	Endodontic disease detection: digital PA vs. CBCT	Addresses disease detection broadly; does not specifically assess postoperative EMS healing

EMS = endodontic microsurgery; CBCT = cone-beam computed tomography; PA = periapical radiography.
